# Supplementary material for: Complex‐centric proteome profiling by SEC‐SWATH‐MS
Source: Mol Syst Biol. 2019 Jan 14;15(1):e8438. doi: 10.15252/msb.20188438 (PMC6346213; doi:10.15252/msb.20188438)
Supplement: Supplementary file 8 — Dataset EV7 [file MSB-15-e8438-s008.zip › feature_plots_string/Q96I99.pdf]

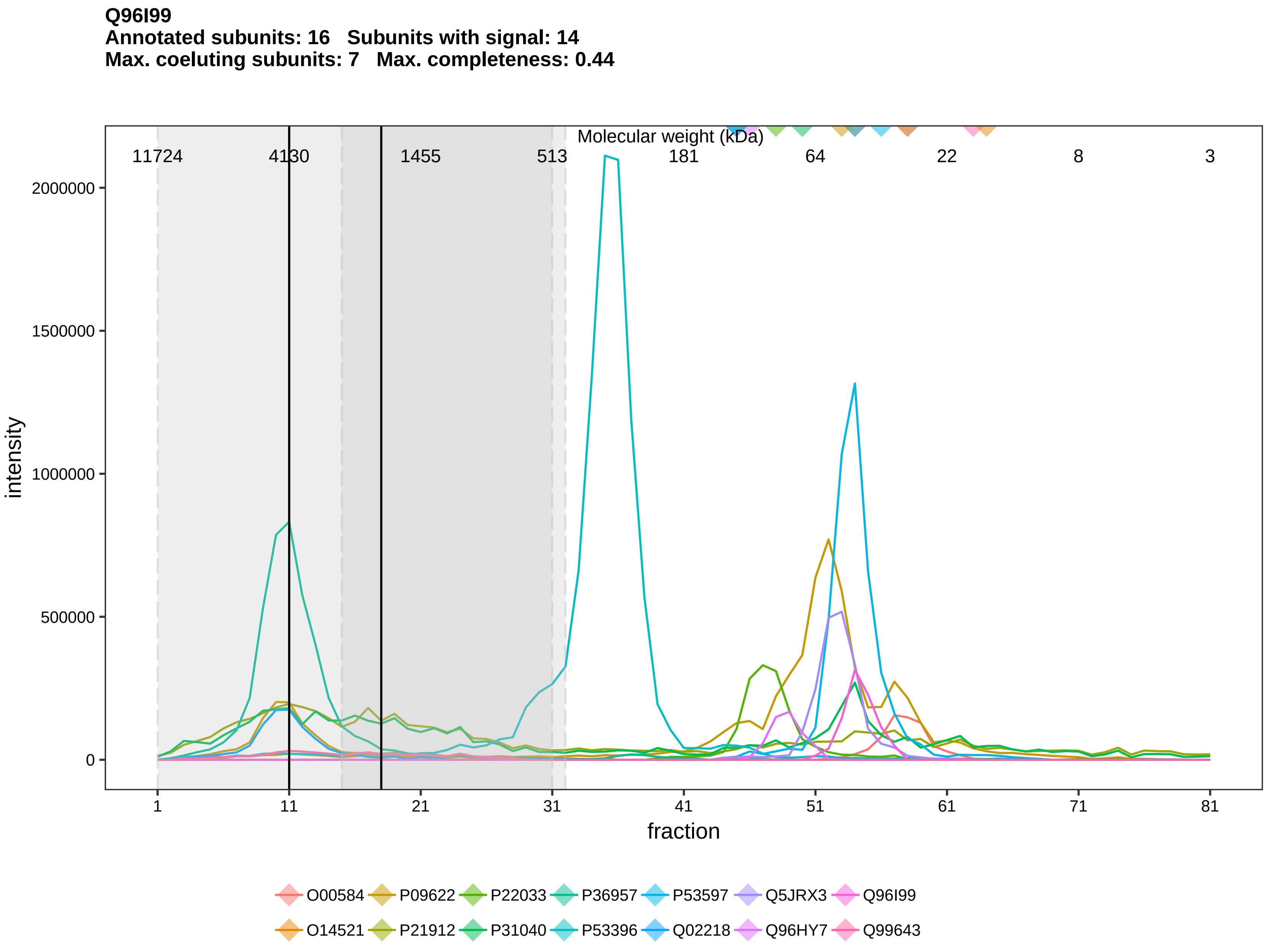
Q96I99
Annotated subunits: 16 Subunits with signal: 14
Max. coeluting subunits: 7 Max. completeness: 0.44
Molecular weight (kDa)
11724
4130
1455
513
181
64
22
8
3
2000000
1500000
1000000
500000
0
intensity
1
11
21
31
41
51
61
71
81
fraction
O00584
P09622
P22033
P36957
P53597
Q5JRX3
Q96I99
O14521
P21912
P31040
P53396
Q02218
Q96HY7
Q99643
